# Is More Always Better for Verbs? Semantic Richness Effects and Verb Meaning

**DOI:** 10.3389/fpsyg.2016.00798

**Published:** 2016-05-31

**Authors:** David M. Sidhu, Alison Heard, Penny M. Pexman

**Affiliations:** Language Processing Laboratory, Department of Psychology, University of Calgary, CalgaryAB, Canada

**Keywords:** semantic richness, verb meaning, past-tense generation, lexical decision, regularity, tense

## Abstract

We examined how several semantic richness variables contribute to verb meaning, across a number of tasks. Because verbs can vary in tense, and the manner in which tense is coded (i.e., regularity), we also examined how these factors moderated the effects of semantic richness. In Experiment 1 we found that age of acquisition (AoA), valence, arousal and embodiment predicted faster response times in LDT. In Experiment 2 we examined a particular semantic richness variable, verb embodiment, and found that it was moderated by tense and regularity. In Experiment 3a we found that AoA predicted faster response times in verb reading. Finally, in Experiment 3b, semantic diversity predicted response times in a past tense generation task, either facilitating or inhibiting responses for regular or irregular verbs, respectively. These results demonstrate that semantic richness variables contribute to verb meaning even when verbs are presented in isolation, and that these effects depend on several factors unique to verbs.

## Introduction

### Semantic Richness Effects

In recent years, a great deal has been learned about representation of word meaning through the study of semantic richness effects in word recognition tasks. The term *semantic richness effects* is used to describe the fact that words that are associated with more semantic information tend to be recognized more efficiently in various lexical and semantic tasks (see Pexman, 2012, for a review). Semantic information can take many forms, as different dimensions of meaning can be derived from different theoretical frameworks for semantic representation. As such, many different semantic richness effects have been observed, and each has been taken as a clue about the nature of semantic representation. For instance, lexical decision responses tend to be faster for words that appear in more diverse linguistic contexts [*semantic diversity* (SemD) effects, Hoffman and Woollams, 2015], suggesting that information about word usage is important to lexical-semantic representation. Lexical decision responses also tend to be faster for words that easily evoke sensory imagery (*imageability* effects, Cortese and Fugett, 2004), or for words with referents with which the human body can easily interact (*body-object interaction* effects, Siakaluk et al., 2008), suggesting that sensorimotor information is important to word meaning. Facilitory semantic richness effects are attributed to stronger semantic activation for semantically richer words, which feeds back to the orthographic and phonological representations, which are presumed to be the focus of lexical decision and pronunciation responses, respectively (Hino and Lupker, 1996; Siakaluk et al., 2008).

Importantly, a number of studies have examined the simultaneous effects of various semantic richness dimensions in lexical-semantic processing and have shown that multiple dimensions can have unique relationships with processing within a single task (Pexman et al., 2008; Yap et al., 2011, 2012). This type of work has lead to the conclusion that semantic representation is multidimensional, incorporating several different types of information, including linguistic or language-based information and experiential or object-based information (e.g., Barsalou et al., 2008; Dove, 2009; Vigliocco et al., 2009; Louwerse, 2010; Binder and Desai, 2011). These inferences have, however, been drawn from research that has focused primarily on concrete nouns, and some of these dimensions are likely not relevant for other word types. A small amount of work has examined semantic richness effects for abstract nouns and has shown that the dimensions of meaning for abstract nouns are somewhat different than those for concrete nouns; for instance, abstract word meaning seems to involve more emotion information than does concrete word meaning (Kousta et al., 2011; Zdrazilova and Pexman, 2013; Siakaluk et al., 2014; Moffat et al., 2015).

Even less attention has been given to verb meaning with the semantic richness approach. Verbs tend to be less concrete than nouns (Allport and Funnell, 1981; Jones, 1985; Bird et al., 2001), and some of the dimensions that are important to noun meaning are not relevant for verbs (e.g., body-object interaction). While concrete nouns refer to objects, verbs refer to actions, states, and relations, and may be inflected for tense, aspect, etc., to agree with the context in which they are used. As such, the dimensions of verb meaning are likely to be somewhat different than those for nouns. It seems possible that much could be learned about those dimensions, and their implications for representation of verb meaning, by extending the semantic richness approach to verbs. This was the purpose of the present study.

Recently, Kemmerer (2015) suggested that the same type of hybrid or multidimensional models that have been used to explain noun meanings (e.g., Binder and Desai, 2011) will also be relevant for verb meanings although, presumably, the particular dimensions involved may differ. Further, these models are flexible and dynamic, so different dimensions of meaning will be more relevant in some contexts than in others. Similar conclusions were reached in a recent study with stroke patients (Desai et al., 2015). Desai et al. (2015) explored representation of verb and noun meanings in a group of stroke patients who had suffered various degrees of motor impairment. Results showed that patients’ degree of impairment was selectively related to their processing of action verbs in lexical decision and also in semantic judgments. For manipulable nouns this relationship was observed only when the task was explicitly semantic (semantic judgments). Desai et al. (2015) concluded that embodied simulations are particularly important to the meanings of action verbs, and that these simulations are modulated by task demands.

In the present study we investigated several candidate dimensions of verb meaning, including semantic richness dimensions derived from emotion information, embodied experience, and linguistic experience, and examined their effects across multiple task contexts. We adopted an approach that has been used in a number of recent megastudies: rather than comparing processing for sets of items that have been matched on specific characteristics, we examined lexical processing for a large number of items, and used regression analyses to examine the effects of candidate semantic richness variables on responses to those items.

If the same kind of flexible, multidimensional models that have been used to describe noun meaning can be applied to verbs, then we expected that some or the entire candidate dimensions examined here would be related to lexical processing. On the other hand, there may be differences in the representation of verb meaning that preclude semantic richness effects for verbs. That is, in lexical decision, naming, and other word recognition tasks, words are presented in isolation. Cordier et al. (2013) posited that when presented in isolation, verbs are impoverished and more ambiguous than nouns, since they are presented without the arguments that come with sentence context. Consequently, Cordier et al. (2013) suggested that verbs generate less semantic activation and less semantic feedback in lexical decision than do nouns. If this is the case, semantic richness effects may be small and difficult to observe for verbs in lexical tasks, and this approach may not reveal dimensions of verb meaning.

### Candidate Dimensions of Verb Meaning

#### Age of Acquisition

Words acquired earlier in life tend to be recognized more efficiently than words acquired later in life, even when related dimensions like frequency and imageability are controlled (e.g., Morrison and Ellis, 1995; Stadthagen-Gonzalez et al., 2004; Cortese and Schock, 2013). This AoA effect has been demonstrated many times, in many different lexical and semantic tasks (for reviews see Ghyselinck et al., 2004; Juhasz, 2005; Johnston and Barry, 2006). One explanation for the effect is that words acquired earlier enjoy richer semantic representations, with more connections to concepts learned later (Steyvers and Tenenbaum, 2005). Indeed, AoA effects do tend to be larger in tasks that involve semantic processing, such as picture naming (Juhasz, 2005). Another explanation is that early-acquired words are represented in a more plastic system, and thus have a stronger influence on network structure (Ellis and Lambon Ralph, 2000). As the system matures, some plasticity is lost, and so later-acquired words have less influence on network structure. One implication of this network plasticity view is that AoA effects are not strictly semantic; instead, AoA affects multiple components of the lexical-semantic system and also the connections between those components (Lambon Ralph and Ehsan, 2006).

Kuperman et al. (2012) collected AoA ratings for over 30,000 words. Using these ratings, Kuperman et al. (2012) reported that AoA had a linear relationship with lexical decision latencies, with faster latencies to words acquired earlier in life. They did not examine this relationship for verbs separately. To our knowledge, AoA effects have been examined separately for verbs in only two studies. In both cases, noun and verb stimuli were presented in mixed lists but responses to the two word types were analyzed separately. All verbs were presented in present tense. Boulenger et al. (2007) compared AoA effects for concrete nouns and action verbs in lexical decision and found that AoA effects were significant only for nouns, once frequency, imageability, and several other factors were controlled. Boulenger et al. (2007) took these results as evidence for differences in the ways that noun and verb meanings are represented. Colombo and Burani (2002) drew a similar conclusion but from the opposite pattern of results, as they found AoA was a stronger predictor of lexical decision latencies for Italian verbs than Italian nouns.

#### Emotion (Valence, Arousal, and Dominance)

Emotion information also seems a good candidate dimension for verb meaning. In a recent study, Warriner et al. (2013) collected ratings of valence (the pleasantness of a stimulus), arousal (the intensity of emotion provoked by a stimulus) and dominance (the degree of control exerted by a stimulus) for 13,915 English lemmas. These included verbs, but only in their base (non-inflected) form. Warriner et al. (2013) assumed that the emotion ratings of present and inflected forms would be similar. Based on their ratings, they concluded that valence and arousal are separate constructs. They did, however, find a strong linear relationship between valence and dominance, suggesting that dominance may not describe a separable affective state. Indeed, most previous studies on emotion in lexical processing have tended to examine only valence and arousal.

Using the Warriner et al. (2013) norms, Kuperman et al. (2014) conducted a megastudy to examine effects of valence and arousal and found that lexical decision and naming responses were faster for positively valenced words and for arousing words. The offered explanation is that negatively valenced words capture more attention than do positive words, due to an automatic vigilance mechanism (Pratto and John, 1991). As a result, processing latencies are often longer for negative words. While this pattern is common, we should note that it is not universal: other patterns of valence and arousal effects have been observed in some studies (e.g., Kousta et al., 2009; Adelman and Estes, 2013; Vinson et al., 2014; Yap and Seow, 2014). Importantly, none of the previous studies on valence or arousal effects have examined verbs separately from nouns.

#### Ambiguity (Semantic Diversity)

Ambiguity is another factor that has not been examined separately for verbs but that could be important to verb meaning. That is, some words have multiple meanings while others have only a single meaning, and this variability has been shown to influence lexical processing. In standard word recognition tasks such as lexical decision, the effect of ambiguity is typically facilitory (e.g., Jastrzembski and Stanners, 1975; Borowsky and Masson, 1996; Hino and Lupker, 1996; cf. Rodd et al., 2002). In contrast, in tasks like semantic relatedness or semantic categorization where word meaning plays an even more central role ambiguity typically has null or inhibitory effects (e.g., Piercey and Joordens, 2000; Pexman et al., 2004; Hargreaves et al., 2011; Yap et al., 2011).

Recently, Hoffman et al. (2011) developed an objective strategy for quantifying the ambiguity of words’ meanings. On the assumption that words that appear in more diverse contexts have more varied meanings, Hoffman et al. (2013) devised the construct of SemD and provided values on this dimension for over 30,000 English words (Hoffman et al., 2013). Hoffman and Woollams (2015) showed that lexical decision responses were faster to high SemD words than to low SemD words and the effect was reversed, with faster responses to low SemD words than to high SemD words, in a semantic relatedness decision task. The assumption is that high SemD words generate noisy semantic representations. These noisy representations facilitate lexical decision responses. When the task requires more fine-grained semantic processing, however, as in semantic relatedness tasks, the noisy semantic representation for high SemD words must be resolved, slowing responses for those items.

#### Sensorimotor (Embodiment and Imageability)

There is now substantial evidence that processing action verbs activates sensory and motor regions of the brain (e.g., Hauk et al., 2004; Pulvermüller et al., 2005), suggesting that sensorimotor information is important to those words’ meanings. Of course, many verbs do not describe specific actions. In a recent study Sidhu et al. (2014) developed a semantic richness dimension called *relative embodiment* that could be applied to all verbs, not just those describing specific overt actions. Note that the term *relative* was used to emphasize that verb embodiment was being measured as a continuous variable, in contrast to studies which had characterized it dichotomously. For the present study, however, this dimension will simply be referred to as *embodiment*. Sidhu et al. (2014) collected embodiment ratings for a large set of verbs, and showed that lexical decision latencies were faster to high embodiment verbs (e.g., *breathe*) than to low embodiment verbs (e.g., *resolve*), even after a number of other lexical and semantic variables were controlled. In addition, action naming latencies (for picture stimuli) and syntactic classification latencies (is it a noun or verb?) were faster for high embodiment verbs. As such, Sidhu et al. (2014) concluded that embodiment is an important dimension of verb meaning.

Imageability refers to the ease with which a word evokes a sensory image. It is a dimension closely related to embodiment. Imageability ratings for verbs are available from norms collected by Chiarello et al. (1999) and Bird et al. (2001). Sidhu et al. (2014) found that for verb stimuli, imageability and embodiment were strongly correlated (at about *r* = 0.70), but that embodiment was a better predictor of lexical processing latencies. Imageability is also highly correlated with concreteness. Colombo and Burani (2002) examined concreteness effects for Italian verbs, but found no significant effect of concreteness on lexical decision latencies.

We examined the effects of these candidate semantic dimensions on lexical processing for verb stimuli under three different task conditions: lexical decision (LDT; Experiment 1), reading (Experiment 3a) and past-tense generation (Experiment 3b). If observed at all, semantic richness effects were expected to be facilitory in these tasks. We also expected differences across LDT and pronunciation tasks, since semantic effects are typically larger in LDT than in naming (Kuperman et al., 2014). Typically, semantic effects are relatively modest in word naming tasks (Balota et al., 2004) since the task does not require participants to identify the meaning of the word.

As mentioned, one of the ways that verb meanings are different from noun meanings is that verb meanings can be inflected for tense. Thus, to fully explore semantic richness effects in verbs, we thought it important to examine these effects beyond the present tense. In Experiments 2 and 3 we explored how verb meaning varies with present vs. past-tense inflection. This also involved an exploration of how semantic richness effects differ with verb regularity. While most verbs can be inflected with the addition of –ed, as in *reach* (*reached*), other verbs are exceptions to the rule, as in *break* (*broke*), *say* (*said*), etc. Most models of past tense generation assume that word knowledge plays a stronger role in past tense generation for irregular verbs than for regular verbs, although there are disagreements about whether this knowledge is lexical (e.g., Pinker and Ullman, 2002) or semantic (e.g., Joanisse and Seidenberg, 1999; Ramscar, 2002; Woollams et al., 2009). That is, the past tense form of irregular verbs is likely to be generated by accessing word-specific knowledge in lexical memory. In contrast, inflection of regular verbs requires less semantic involvement since regular verbs all follow the same rules or procedures for inflection.

Thus, as a strategy to further explore verb meaning, we compared embodiment ratings for regular and irregular verbs in present and past tense in Experiment 2. We selected this particular semantic dimension because previous research has shown that tense can modulate the extent of sensorimotor activation for action verbs. For instance, Candidi et al. (2010) reported stronger activation of hand muscles when reading hand-related action verbs in future tense vs. past tense. Similarly, studies have shown that as temporal distance increases, for instance, from the present into the past, representations become less concrete (for a review see Trope and Liberman, 2010). While the present implies direct experience, the past is necessarily distinct from direct experience. Thus, it seemed possible that embodiment ratings might be lower for verbs in past tense than in present tense.

Further, in Experiment 3 we included both a reading (pronunciation) and a past tense generation task, and examined potential interactions of semantic richness variables with verb regularity in these pronunciation tasks. While past tense generation latencies have not often been examined in healthy adult populations (cf. Butler et al., 2012; Cohen-Shikora et al., 2013), we saw the task as an opportunity to further explore verb meaning, particularly for irregular verbs. Since semantic processing is assumed to be more relevant to past tense generation for irregular verbs than regular verbs, it seemed possible that semantic richness effects might interact with regularity in that task. This would be consistent with a study by Butler et al. (2012), which found that the effects of imageability on past tense generation were moderated by verb regularity.

We considered it important to ensure that participants were processing the verb meanings of our stimuli. Many verbs also have noun meanings (e.g., *play*) and if the verbs were presented in a mixed list with noun stimuli, or were presented without cueing to interpret the word as a verb, the noun meanings might be prioritized. As such, in all experiments, participants were only presented with verb stimuli and were prompted to process the items as verbs. That is, in Experiment 1 the word “to” appeared in front of each letter string presented for lexical decisions, while in Experiments 2 and 3 participants were told that they would only be presented with verbs, and would be asked to rate (Experiment 2), read (Experiment 3a) or generate the past tense (Experiment 3b) for each verb.

In summary, we expected that if semantic richness effects are observed for verbs, those effects might not be large in tasks that depend primarily on lexical processing (LDT, Experiment 1; verb reading, Experiment 3a). Hence, we included tasks that require more extensive semantic processing, and where verb-specific factors like regularity and tense might be influential (ratings, Experiment 2; past tense generation, Experiment 3b), in order to gain additional insights about verb meaning.

## Experiment 1

### Method

This and subsequent studies were carried out in accordance with the University of Calgary Ethics Committee, with written informed consent from all subjects. All subjects gave written informed consent in accordance with the Declaration of Helsinki.

The LDT data obtained for Experiment 1 were from Sidhu et al. (2014; Experiment 1). That study, and each of the following experiments, were approved by the University of Calgary Conjoint Faculties Research Ethics Board. In the Sidhu et al. (2014) study, 30 undergraduate participants made LD responses to 400 verbs and 400 non-words. Each trial began with an asterisk, which then was replaced by a letter string after 1500 ms. Verbs were presented in their infinitive form (e.g., *to leap*), and non-words were also preceded by the word *to*. Sidhu et al. (2014) analyzed LDT latencies only for correct trials, and excluded trials on which latencies were more than 2.5 *SD* from a participant’s mean. In addition, eight verbs were excluded from the analyses due to high error rate, leaving a total of 392 verbs. These data were the starting point for the present analysis. For a small number of these items, however, values for valence, arousal, and dominance were not available in the Warriner et al. (2013) norms, and/or SemD values were not available from the Hoffman et al. (2013) norms. As such, the LDT analyses below include 369 verbs. Descriptive characteristics for this subset of the Sidhu et al. (2014) items are presented in **Table 1**.

**Table 1:** Mean descriptive statistics (standard deviations in parentheses; minimum and maximum values below) for verb stimuli in Experiments 1 (LDT), 2 (Embodiment Ratings), and 3 (Reading and Past Tense Generation).

	Experiment 1	Experiment 2		Experiment 3	
Variable	(*n* = 369)	Regular (*n* = 603)	Irregular (*n* = 80)	Regular (*n* = 316)	Irregular (*n* = 79)
Log Frequency	8.67 (1.81)	8.27 (1.89)	10.44 (2.00)	8.40 (1.66)	10.53 (1.86)
	[5.04, 14.92]	[2.94, 13.87]	[4.38, 14.92]	[5.04, 13.14]	[6.29, 14.92]
OLD	1.91 (0.54)	2.01 (0.56)	1.52 (0.46)	1.99 (0.52)	1.49 (0.41)
	[1.00, 4.25]	[1.00, 4.35]	[1.00, 3.25]	[1.00, 4.25]	[1.00, 2.65]
PLD		1.89 (0.68)	1.30 (0.46)	1.88 (0.63)	1.27 (0.43)
		[1.00, 4.45]	[1.00, 2.75]	[1.00, 4.25]	[1.00, 2.75]
Age of Acquisition (AoA)	7.81 (2.37)	8.22 (2.33)	5.49 (1.78)	8.22 (2.22)	5.38 (1.68)
	[2.78, 13.29]	[2.89, 14.00]	[2.78, 10.95]	[3.26, 13.29]	[2.78, 10.95]
Valence	5.12 (1.34)	5.14 (1.41)	5.40 (1.34)	5.07 (1.33)	5.41 (1.31)
	[1.48, 7.95]	[1.48, 8.00]	[2.17, 7.73]	[1.48, 7.95]	[2.17, 7.73]
Arousal	4.26 (0.94)	4.28 (0.93)	4.07 (0.74)	4.28 (0.97)	4.10 (0.74)
	[2.29, 7.05]	[2.29, 7.05]	[2.60, 6.33]	[2.29, 7.05]	[2.60, 6.33]
Dominance	5.44 (0.97)	5.49 (1.01)	5.50 (0.94)	5.43 (0.98)	5.51 (0.91)
	[2.57, 7.72]	[2.37, 7.72]	[3.00, 7.26]	[2.57, 7.72]	[3.00, 7.26]
Semantic diversity (SemD)	1.81 (0.26)	1.78 (0.27)	1.91 (0.23)	1.80 (0.25)	1.90 (0.24)
	[0.47, 2.35]	[0.39, 2.28]	[1.40, 2.35]	[0.47, 2.26]	[1.40, 2.35]
Imageability	4.25 (1.00)	415.46 (98.73)	440.81 (103.54)	417.84 (98.12)	444.95 (101.29)
	[154, 643]	[154, 643]	[204, 639]	[154, 643]	[204, 639]
Embodiment (Present)	4.03 (1.32)	3.93 (1.23)	4.51 (1.28)	3.91 (1.29)	4.46 (1.17)
	[1.97, 6.58]	[1.76, 6.77]	[2.13, 6.73]	[1.97, 6.58]	[2.58, 6.48]
Embodiment (Past)		3.71 (1.23)	4.05 (1.28)	3.80 (1.42)	4.12 (1.31)
		[1.63, 6.77]	[1.97, 6.57]	[1.67, 6.73]	[1.97, 6.57]
LDT latency (ms)	605.50 (54.64)				
	[510.57, 874.65]				
LDT latency (*z*-score)	-0.11 (0.32)				
	[-0.70, 1.08]				
Reading latency (ms)				590.31 (36.44)	573.24 (31.84)
				[509.20, 705.82]	[510.57, 715.29]
Reading latency (*z*-score)				-0.04 (0.26)	-0.16 (0.25)
				[-0.58, 0.87]	[-0.70, 0.44]
Past tense generation latency (ms)				785.51 (69.46)	944.05 (94.26)
				[650.67, 1055.41]	[744.47, 1151.87]
Past tense generation latency (*z*-score)				-0.20 (0.25)	0.36 (0.34)
				[-0.69, 0.77]	[-0.37, 1.46]

*OLD, orthographic Levenshtein distance; PLD, phonological Levenshtein distance (Yarkoni et al., 2008); LDT, lexical decision task.*

### Results

The variables in the analysis were divided into two clusters: control variables and semantic variables. Control variables included the lexical variables that have been shown to be the most important predictors of LDT performance (Brysbaert et al., 2011): frequency (log transformed HAL word frequency, Lund and Burgess, 1996) and orthographic similarity (orthographic Levenshtein distance; OLD, Yarkoni et al., 2008). Semantic variables were AoA (Kuperman et al., 2012), valence, arousal, and dominance (Warriner et al., 2013), SemD (Hoffman et al., 2013), imageability (Chiarello et al., 1999; Bird et al., 2001) and embodiment (Sidhu et al., 2014). We also included regularity as a predictor in this latter cluster of variables.

Correlations between variables are presented in **Table 2**. The relationships between most of the richness variables were modest, suggesting that these dimensions capture somewhat different aspects of meaning. In contrast, the correlations between valence and dominance, and imageability and embodiment, were relatively high. To avoid multicollinearity we included valence (but not dominance) and embodiment (but not imageability) in the regression analyses presented below, as the previous literature suggested these were the more likely predictors of LDT latencies, and these variables had the higher raw correlations with LDT latencies (and also with the criterion variables in Experiment 3).^1^

**Table 2:** Correlations between predictor variables and dependent measures in Experiment 1 (LDT).

Variable	1	2	3	4	5	6	7	8	9	10
(1) Frequency	–									
(2) OLD	-0.30***	–								
(3) Regularity	0.37***	-0.35***	–							
(4) AoA	-0.53***	0.50***	-0.43***	–						
(5) Valence	0.23***	-0.08	0.08	-0.19***	–					
(6) Arousal	-0.10	0.00	-0.06	-0.04	-0.34***	–				
(7) Dominance	0.22***	-0.01	0.03	-0.08	0.72***	-0.25***	–			
(8) SemD	0.51***	0.04	0.12*	-0.08	0.12*	-0.17**	0.17**	–		
(9) Imageability	-0.06	-0.24***	0.17**	-0.51***	-0.03	0.27***	-0.12*	-0.40***	–	
(10) Embodiment	0.04	-0.37***	0.22**	-0.55***	-0.01	0.27***	-0.10	-0.27***	0.70***	–
(11) LDT latency	-0.63***	0.37***	-0.31***	0.57***	-0.21***	-0.05	-0.20***	-0.25***	-0.19***	-0.28***

*OLD, orthographic Levenshtein distance; AoA, age of acquisition; SemD, semantic diversity; LDT, lexical decision task.*

*^∗^*p* < 0.05; ^∗∗^*p* < 0.01; ^∗∗∗^*p* < 0.001.*

While we predicted that valence would be linearly related to LDT latencies, we also wanted to evaluate the possibility that valence might have the inverted U-shaped relationship with LDT latencies (faster latencies for both positive and negative words than for neutral words) observed in some previous studies (e.g., Yap and Seow, 2014). As such, we also coded valence in terms of extremity (distance from scale mid-point, see also Adelman and Estes, 2013) but found that while the correlation between valence and LDT latencies was significant (*r* = -0.21, *p* < 0.001), the correlation between extremity and LDT latencies was not (*r* = -0.03, *p* = 0.61) and so we included valence and not extremity in our analyses.

LDT latencies were standardized as *z*-scores since these minimize the influence of a participant’s processing speed and variability (Faust et al., 1999). All predictor variables were centered. A hierarchical regression analysis was conducted on the standardized LDT latencies. Control variables were entered in step 1 and semantic variables in step 2, and the regression results are presented in **Table 3**. Results for the control variables showed the typical effects: word frequency and OLD were both significant predictors of LDT latencies for verbs, with faster latencies for more frequent and less orthographically distinct verbs. Together the semantic variables accounted for a small but significant amount of additional variability, and significant unique relationships with LDT latencies were observed for AoA, valence, arousal and embodiment. That is, LDT latencies were faster for verbs acquired earlier in life, and for verbs with more positive, arousing, and embodied meanings. The unique relationship between verb regularity and LDT latencies was not significant, likely because verbs were presented in present tense and no inflection was required for the response. The relationship between SemD and LDT latencies was also not significant. This finding is consistent with the results of Taikh et al. (2015) for noun stimuli, where SemD did not have a unique relationship to LDT latencies once other lexical and semantic variables were included in the analysis.

**Table 3:** Regression coefficients from item-level regression analyses for standardized LDT latencies, Experiment 1.

Variable	*B*	*SEB*	β	*sr*	*R*^2^	Δ *R*^2^
Step 1 (Control variables)					0.41^∗∗∗^	0.41^∗∗∗^
Frequency	-0.10	0.01	-0.55	-0.53***		
OLD	0.12	0.03	0.20	0.19***		
Step 2 (Semantic variables)					0.47^∗∗∗^	0.06^∗∗^
Regularity	0.00	0.04	0.00	0.00		
Age of acquisition (AoA)	0.02	0.01	0.18	0.11**		
Valence	-0.02	0.01	-0.08	-0.08*		
Arousal	-0.03	0.01	-0.09	-0.08*		
Semantic Diversity (SemD)	0.01	0.06	0.00	0.00		
Embodiment	-0.03	0.01	-0.11	-0.08*		

*OLD, orthographic Levenshtein distance. ^∗^*p* < 0.05; ^∗∗^*p* < 0.01; ^∗∗∗^*p* < 0.001.*

Thus, several semantic richness effects were observed in LDT, consistent with the notion that even when presented in isolation, verbs do generate measurable semantic feedback. These results suggest, further, that semantic representations for verbs are multidimensional (Kemmerer, 2015), and include episodic, emotional, and sensorimotor information. We next investigated two factors that could be important for verb meaning and could modulate richness effects: tense and regularity. We did so in a ratings task that encouraged participants to consider word meanings more specifically.

## Experiment 2

The purpose of Experiment 2 was to explore effects of tense and regularity on verb meaning. We examined these effects on a dimension of meaning that might be expected to vary as a function of tense: embodiment. That is, the present tense implies direct experience, which may lead to higher embodiment ratings than for the same verbs presented in past tense.

### Method

#### Participants

Participants in Experiment 2 were 120 undergraduate students at the University of Calgary who participated for bonus credit in a Psychology course. All participants had normal or corrected-to-normal vision, and reported English proficiency.

#### Stimuli and Procedure

The stimuli for Experiment 2 were the 687 verbs from the Sidhu et al. (2014) embodiment ratings. These were divided into four lists. Each participant was presented with the items from two of the four lists, with one list presented in present tense and the other presented in past tense. Present and past tense verbs were randomly intermixed for each participant and presented for ratings. Ratings instructions were the same as those used in Sidhu et al. (2014), and are presented in the current Appendix A.

Verbs were presented for ratings in an on-line study using the survey tool Survey Monkey^2^. Participants were asked to rate each verb for relative embodiment using a 1–7 scale, and made their selection via mouse click.

The data for one participant who used only two points on the rating scale were not included in the analysis. In addition, the ratings data for four of the verbs were not usable: ratings for *found* were not analyzed because this word takes the same form as the past tense of *find*, which was also included in the survey, and the past tense versions of three other verbs were misspelled in the ratings task so the resulting ratings were not analyzed. The descriptive characteristics of the remaining 683 items are presented in **Table 1**. The correlation between present and past tense ratings for these 683 items was *r* = 0.92, which is consistent with Warriner et al.’s (2013) assumption that ratings for base form meaning would be very similar to ratings for inflected form meaning. The correlation between the present tense ratings collected here and the original Sidhu et al. (2014) present tense ratings was also *r* = 0.92, suggesting reasonable reliability.

We then examined the embodiment ratings with a 2 (tense: present, past) × 2 (regularity: regular, irregular) ANOVA. Results showed a significant interaction between tense and regularity [*F*(1,681) = 15.13, *MSE* = 0.12, *p* < 0.001, η^2^= 0.02], such that embodiment ratings were higher for present tense irregular verbs (*M* = 4.52, *SD* = 1.28) than for past tense irregular verbs (*M* = 4.06, *SD* = 1.28), and this tense difference was attenuated for present tense regular verbs (*M* = 3.93, *SD* = 1.23) and past tense regular verbs (*M* = 3.71, *SD* = 1.23). The main effects of tense [*F*(1,681) = 131.76, *MSE* = 0.12, *p* < 0.001, η^2^= 0.16], and regularity [*F*(1,681) = 10.47, *MSE* = 2.95, *p* = 0.001, η^2^= 0.01], were also significant. Thus, consistent with predictions, embodiment ratings tended to be lower for verbs presented in past tense. This difference was more pronounced for irregular verbs.

The descriptive statistics presented in **Table 1** show, however, that frequency (log transformed HAL word frequency, Lund and Burgess, 1996) was significantly higher for irregular verbs (*M* = 10.44, *SD* = 2.00) than for regular verbs [*M* = 8.27, *SD* = 1.89; *t*(681) = 9.60, *p* < 0.001]. We conducted a supplementary analysis to investigate whether this might have contributed to the effects of tense and regularity observed in the embodiment ratings. We examined embodiment ratings with a 2 (tense: present, past) × 2 (regularity: regular, irregular) ANCOVA, including verb frequency as a covariate. Results indicated that frequency was not significantly related to embodiment [*F*(1,680) = 3.21, *p* = 0.07], and that the interaction of tense and regularity [*F*(1,680) = 7.65, *MSE* = 0.12, *p* = 0.006, η^2^= 0.01] and main effect of regularity [*F*(1,680) = 13.40, *MSE* = 2.94, *p* < 0.001, η^2^= 0.02] were still significant. The main effect of tense, however, was not significant [*F*(1,680) = 0.97, *p* = 0.32], These results suggest that differences in frequency may explain some of the effects of tense on embodiment.

We next investigated semantic richness effects for verbs under different task demands. In Experiment 3a we used a verb reading (pronunciation) task and in Experiment 3b we used a past-tense generation task. We increased the number of irregular verbs included in the stimulus list that had been presented in Experiment 1 and, in both Experiments 3a and 3b, tested for interactions of regularity with the semantic richness variables. We expected that interactions with regularity would be most likely in Experiment 3b since in the past-tense generation task semantic processing was expected to be relatively more extensive for irregular verbs.

## Experiment 3a

### Method

#### Participants

Participants in Experiment 3a were 30 undergraduate students at the University of Calgary who participated for bonus credit in a Psychology course. All participants had normal or corrected-to-normal vision, and reported English proficiency.

#### Stimuli and Procedure

The stimuli for Experiment 3a were the same as described for Experiment 1, plus an additional 22 irregular verbs that had not been presented in Experiment 1. This exhausted the pool of irregular verbs from the original Sidhu et al. (2014) ratings. In total, 422 verb stimuli were presented in Experiment 3a.

Participants were seated in front of a computer, and on each trial a verb was presented on the screen in present tense. Participants were asked to simply say the verb out loud as quickly and as accurately as they could. Stimuli were presented to each participant in a different random order. Reading latencies were detected via a microphone connected to a voice key. Reading responses were also recorded for later coding of response errors.

### Results

Pronunciation latencies were excluded from analyses for trials on which participants failed to trigger the voice key (1.42% of trials), stuttered (0.26% of trials), or mispronounced the target (1.76% of trials). We also excluded reading latencies faster than 200 ms (0.36% of trials), slower than 3000 ms (0.77% of trials), or greater than 2.5 SD from the participant’s mean (2.56% of trials). There were too few genuine mispronunciation errors to warrant analysis, so only the latency data were analyzed.

We had a complete set of predictors for 395 items. The descriptive characteristics for these items are presented in **Table 1**. As in Experiment 1, we first examined correlations between variables (**Table 4**) and then used hierarchical linear regression analyses to examine the influence of the semantic richness variables on reading latencies (**Table 5**). All predictor variables were centered and correct reading latencies were standardized. Since the task involved vocal responses, on step 1 of the regression analysis we included a set of variables that code for item differences in initial phoneme onsets. Dichotomous variables were used to code the initial phoneme of each word (1 = presence of a feature; 0 = absence of a feature) on 13 features: affricative, alveolar, bilabial, dental, fricative, glottal, labiodental, liquid, nasal, palatal, stop, velar, and voiced (Balota et al., 2004). On step 2 we included the key lexical variables: log transformed HAL word frequency (Lund and Burgess, 1996) and phonological Levenshtein distance (PLD, Yarkoni et al., 2008). We used PLD here (instead of OLD, which was included in Experiment 1) because, while OLD and PLD tend to be highly correlated, phonological neighborhoods tend to be more important to pronunciation tasks. We also included phonological consistency (Ziegler et al., 1997) as this also tends to predict responses in pronunciation tasks. On step 3 we included the same semantic variables as in Experiment 1, but here we added terms for regularity (1 = irregular; 0 = regular) and the interactions of the semantic richness variables with regularity.

**Table 4:** Correlations between predictor variables and dependent measures in Experiment 3a and 3b (Reading and Past Tense Generation).

Variable	1	2	3	4	5	6	7	8	9	10	11	12	13
(1) Frequency	–												
(2) PLD	-0.32***	–											
(3) Phonological consistency	0.02	0.12*	–										
(4) Regularity	0.44***	-0.38***	0.01	–									
(5) AoA	-0.58***	0.51***	0.01	-0.47***	–								
(6) Valence	0.25***	-0.05	-0.02	0.10*	-0.21***	–							
(7) Arousal	-0.11*	-0.01	0.04	-0.08	-0.02	-0.35***	–						
(8) Dominance	0.22***	0.02	0.05	0.03	-0.09	0.72***	-0.26***	–					
(9) SemD	0.52***	0.08	-0.01	0.17**	-0.08	0.14**	-0.18***	0.19***	–				
(10) Imageability	-0.06	-0.26***	-0.05	0.10*	-0.46***	-0.03	0.27***	-0.12*	-0.45***	–			
(11) Embodiment – Present	0.03	-0.34***	-0.06	0.16**	-0.51***	0.00	0.26***	-0.08	-0.31***	0.70***	–		
(12) Embodiment – Past	-0.04	-0.32***	-0.06	0.07	-0.48***	-0.04	0.23***	-0.12*	-0.33***	0.71***	0.94***	–	
(13) Reading latency	-0.45***	0.39***	0.09	-0.17**	0.45***	-0.09	-0.05	-0.04	-0.05	-0.15**	-0.22***	-0.18***	–
(14) PTG latency	0.20***	-0.36***	0.15**	0.64***	-0.33***	0.04	-0.05	-0.03	0.06	0.08	0.17**	0.11*	0.11^∗^

*PLD, phonological Levenshtein distance; AoA, age of acquisition; SemD, semantic diversity; PTG, past tense generation.*

*^∗^*p* < 0.05; ^∗∗^*p* < 0.01; ^∗∗∗^*p* < 0.001.*

**Table 5:** Regression coefficients from item-level regression analyses for standardized reading and past tense generation latencies, Experiments 3a and 3b.

Variable	*B*	*SEB*	β	*sr*	*R*^2^	Δ *R*^2^
**Experiment 3a (Verb reading**
Step 1 (Onsets)					0.19^∗∗∗^	0.19^∗∗∗^
Step 2 (Control variables)					0.44^∗∗∗^	0.25^∗∗∗^
Frequency	-0.05	0.01	-0.35	-0.33***		
PLD	0.12	0.02	0.29	0.26***		
Phonological consistency	-0.01	0.02	-0.02	-0.02		
Step 3 (Semantic variables)					0.49^∗∗∗^	0.05^∗∗∗^
Regularity	0.02	0.04	0.03	0.02		
Age of acquisition (AoA)	0.02	0.01	0.22	0.12**		
Valence	-0.00	0.01	-0.01	-0.01		
Arousal	-0.01	0.01	-0.03	-0.02		
Semantic diversity (SemD)	0.11	0.05	0.10	0.07		
Embodiment	-0.00	0.01	-0.02	-0.01		
Age of acquisition × Regularity	-0.03	0.02	-0.12	-0.05		
Valence × Regularity	0.03	0.02	0.06	0.04		
Arousal × Regularity	0.02	0.04	0.02	0.02		
Semantic diversity × Regularity	-0.07	0.12	-0.03	-0.02		
Embodiment × Regularity	0.03	0.03	0.05	0.04		
**Experiment 3b (Past tense generation)**
Step 1 (Onsets)					0.19^∗∗∗^	0.19^∗∗∗^
Step 2 (Control variables)					0.32^∗∗∗^	0.13^∗∗∗^
Frequency	0.03	0.01	0.15	0.14**		
PLD	-0.15	0.03	-0.27	-0.25***		
Phonological consistency	0.12	0.03	0.16	0.16***		
Step 3 (Semantic variables)					0.54^∗∗∗^	0.22^∗∗∗^
Regularity	0.41	0.06	0.46	0.26***		
Age of acquisition (AoA)	0.01	0.01	0.05	0.03		
Valence	-0.00	0.01	-0.00	-0.00		
Arousal	-0.01	0.02	-0.03	-0.02		
Semantic diversity (SemD)	-0.01	0.07	-0.01	-0.01		
Embodiment	0.02	0.02	0.07	0.04		
Age of acquisition × Regularity	-0.04	0.02	-0.13	-0.06		
Valence × Regularity	-0.00	0.03	-0.00	-0.00		
Arousal × Regularity	0.05	0.05	0.05	0.04		
Semantic diversity × Regularity	0.30	0.15	0.10	0.08*		
Embodiment × Regularity	-0.03	0.03	-0.04	-0.03		

*PLD, phonological Levenshtein distance. ^∗^*p* < 0.05; ^∗∗^*p* < 0.01; ^∗∗∗^*p* < 0.001.*

The regression analysis showed that the onsets and control variables explained a substantial amount of variance in verb reading latencies. Responses were faster for more frequent verbs and less phonologically distinct verbs. A small but significant amount of variance was explained by the semantic variables. Although several of the semantic richness variables had significant relationships with reading latencies in the raw correlations, only AoA was significant once the control variables were entered in the regression analysis. That is, reading latencies were faster for verbs acquired earlier in life. While the AoA effect in pronunciation has been demonstrated in several previous studies (e.g., Morrison and Ellis, 1995), to our knowledge this is the first time it has been demonstrated specifically for verbs.

The regression analyses also showed that regularity was not significantly related to reading latencies, and none of the terms for interactions of semantic richness variables with regularity were significant. Our expectation, however, was that these predictors might be significantly related to past tense generation latencies, in Experiment 3b.

## Experiment 3b

### Method

#### Participants

Participants in Experiment 3b were 30 undergraduate students at the University of Calgary who participated for bonus credit in a Psychology course. All participants had normal or corrected-to-normal vision, and reported English proficiency.

#### Stimuli and Procedure

The stimuli and procedure for Experiment 3b were the same as described for Experiment 3a except that in this task participants were asked to pronounce the past tense form of each verb presented, as quickly and as accurately as possible.

### Results

Past tense generation latencies were excluded from analyses for trials on which participants failed to trigger the voice key (1.82% of trials), stuttered (0.23% of trials), or mispronounced the target (3.30% of trials). In addition, participants occasionally produced a regularized past tense version of irregular verbs; these comprised an additional 1.69% of trials (8.58% of irregular trials) and were also excluded from latency analyses. We further excluded latencies faster than 200 ms (0.35% of trials), slower than 3000 ms (2.53% of trials), or greater than 2.5 *SD* from the participant’s mean (2.87% of trials). As in Experiment 3a, there were too few mispronunciation errors to warrant analysis, so only the latency data were analyzed.

The raw correlation results in **Table 4** showed that the various predictors tended to have opposite relationships with latencies in the verb reading (Experiment 3a) and past tense generation (Experiment 3b) tasks. The variables that were facilitory for verb reading tended to be inhibitory for past tense generation, and vice versa: for instance, in raw correlations, frequency, regularity, and embodiment were all facilitory for verb reading but inhibitory for past tense generation. Latencies for the two tasks were only modestly related. These results suggest that very different processes are at work in the two tasks.

We again used hierarchical linear regression analyses (**Table 5**), here to examine the influence of the semantic richness variables on past tense generation latencies. The predictors were the same as those used in Experiment 3a. As mentioned, the raw correlation between present and past tense embodiment ratings was high. We opted to use present tense embodiment ratings in the regression analysis as this embodiment variable had higher raw correlations with past tense generation performance than did the past tense embodiment ratings. The regression results showed that phonological consistency was a significant predictor of past tense generation latencies, with slower past tense generation latencies for phonologically inconsistent verbs. This is presumably because competition from a verb’s consistent body neighbors delays activation of the phonological code.

In the regression analyses, regularity had a strong positive relationship with past tense generation latencies. The nature of the regularity effect was that past tense generation latencies were much faster for regular verbs (*M* = 785.51, *SD* = 69.46) than for irregular verbs (*M* = 944.05, *SD* = 94.26). This is consistent with the results of previous past-tense generation studies (Woollams et al., 2009; Cohen-Shikora and Balota, 2013), and suggests that past tense generation for irregular verbs involves additional processing that is not necessary for past tense generation of regular verbs. Other aspects of our results suggest that the additional processing for irregular verbs likely involves semantic processing. That is, the semantic variable that had a significant unique relationship with past-tense generation latencies in the regression analysis was the interaction of SemD and regularity. The nature of the interaction between SemD and regularity was explored with separate correlation analyses for regular and irregular verbs (**Table 6**). These showed that the relationship between SemD and past-tense generation latencies was negative for regular verbs and positive for irregular verbs. Thus, for regular verbs past tense generation latencies were facilitated by SemD, while for irregular verbs past tense generation latencies were inhibited by SemD. We give this finding fuller consideration in Section “General Discussion.”

**Table 6:** Correlations between predictor variables and dependent measures in Experiment 3a and 3b (Reading and Past Tense Generation).

Variable	1	2	3	4	5	6	7	8	9	10	11	12	13
(1) Frequency	–	-0.37**	0.13	-0.66***	0.47***	-0.25*	0.40***	0.59***	-0.27**	-0.11	-0.19*	-0.36**	0.25*
(2) PLD	-0.16*	–	0.08	0.35**	-0.13	0.07	-0.13	-0.03	-0.29**	-0.42***	-0.41***	0.17	-0.25*
(3) Phonological Cons.	-0.02	0.15**	–	0.02	0.09	0.11	0.11	0.03	-0.11	-0.02	-0.07	0.15	0.31**
(4) AoA	-0.44***	0.43***	0.02	–	-0.42***	0.24*	-0.32**	-0.19	-0.21	-0.36**	-0.35**	0.36**	-0.07
(5) Valence	0.17**	0.01	-0.05	-0.14***	–	-0.49***	0.79***	0.15	-0.05	-0.04	-0.08	-0.14	-0.01
(6) Arousal	-0.05	-0.06	0.03	-0.10	-0.32***	–	-0.38**	-0.16	0.12	0.14	0.15	0.12	0.08
(7) Dominance	0.20***	0.06	0.04	-0.05	0.71***	-0.24***	–	0.27*	-0.12	-0.08	-0.12	-0.02	-0.02
(8) SemD	0.48***	0.11***	-0.02	-0.01	0.11	-0.17***	0.17**	–	-0.55***	-0.40**	-0.36**	-0.19	0.21*
(9) Imageability	-0.08	-0.23***	-0.04	-0.54***	-0.04	0.30***	-0.12*	-0.41***	c–	0.70***	0.71***	-0.03	-0.17
(10) Embodiment - Present	-0.03	-0.31***	-0.07	-0.53***	-0.01	0.30***	-0.09	-0.28***	0.69***	–	0.91***	-0.01	-0.01
(11) Embodiment - Past	-0.06	-0.32***	-0.06	-0.54***	-0.03	0.26***	-0.12*	-0.29***	0.70***	0.95***	–	0.01	-0.09
(12) Reading latency	-0.44***	0.43***	0.08	0.48***	-0.07	-0.10	-0.04	-0.09	-0.17**	-0.26***	-0.23***	–	0.23*
(13) PTG latency	-0.23***	-0.11*	0.15**	0.00	-0.05	-0.01	-0.08	-0.21**	0.07	0.10	0.12*	0.30***	–

*Correlations for irregular verbs are presented above the diagonal and correlations for regular verbs are presented below the diagonal. PLD, phonological Levenshtein distance; Phonological Cons, phonological consistency; AoA, age of acquisition; SemD, semantic diversity; PTG, past tense generation. ^∗^*p* < 0.05; ^∗∗^*p* < 0.01; ^∗∗∗^p < 0.001.*

The correlation results presented in **Table 6** provide more insight about the inhibitory effect of frequency found on the second step of the regression analysis. That is, for regular verbs the relationship between frequency and past-tense generation latencies was in the typical facilitory direction, while for irregular words there was an inhibitory relationship between frequency and past-tense generation latencies. That is, past-tense generation latencies were slower for more frequent irregular verbs, and this seemed to drive the overall results in the main regression analysis. A similar inhibitory effect of frequency for past tense generation latencies for Dutch verbs was reported by Tabak et al. (2005). One explanation is that in the past tense generation task, because the verb is presented in present form (e.g., *bring*), participants automatically generate the phonology of the present tense form, which produces response conflict with the inflected form (*brought*; Woollams et al., 2009). The response conflict is greater for irregular verbs, where the phonology of present and past tense forms are more distinct, and is also greater for more frequent verbs, for which the phonology of the present tense form is generated more readily. Thus, past tense generation responses are slower for more frequent verbs. These findings suggest that in the past tense generation task, response conflict between what is presented and what must be produced drives some of the effects observed.

## General Discussion

The purpose of the present study was to investigate semantic richness effects for verb stimuli in order to test several candidate dimensions of verb meaning. We presented only verb stimuli and encouraged participants to treat the stimuli as verbs. Although there has been some suggestion that verbs presented in isolation will generate relatively little semantic feedback (Cordier et al., 2013), we found significant semantic richness effects in LDT for dimensions that tap several different aspects of verb meaning: AoA, valence, arousal, and embodiment. Under different task demands, we found that only AoA was related to verb reading and that SemD tended to be facilitory for regular verbs but inhibitory for irregular verbs in a past-tense generation task. As such, our results are consistent with assertions that verb meaning is multidimensional and flexible (Desai et al., 2015; Kemmerer, 2015). Our findings suggest that dimensions of verb meaning include episodic, emotional, and sensorimotor information, and the influence of these and other unexplored factors will depend on the context in which verb meaning is processed.

We observed facilitory frequency effects and inhibitory AoA effects in LDT and in verb reading but not in past tense generation. This confirms that these are the tasks where lexical processes dominate. Additionally, the fact that AoA effects were observed in these tasks provides insight about the locus of AoA effects. That is, AoA effects were observed in lexical tasks that involve several components: orthographic, phonological (particularly in verb reading) and semantic (particularly in LDT) processing. This is consistent with the claim that there is a broad basis for AoA effects. That is, processing is facilitated for early acquired words because these have a stronger influence on system structure than do later-acquired words.

Our results are also consistent with previous studies (e.g., Balota et al., 2004) in showing that semantic variables explained more variance in lexical decision than in pronunciation tasks. The past-tense generation task, however, presented an opportunity to examine somewhat more extensive semantic processing than is involved in most pronunciation tasks, and to explore a central distinction for verb stimuli, by examining latencies for regular and irregular verbs. As mentioned, research with nouns suggests that ambiguity tends to function differently than other semantic richness effects. Most richness dimensions are facilitory in both lexical and semantic tasks (Pexman et al., 2014). In contrast, the effects of variables like SemD that capture ambiguity are typically facilitory in LDT, but null or inhibitory in semantic tasks (e.g., Hargreaves et al., 2011; Yap et al., 2011, 2012; Hoffman and Woollams, 2015). The explanation for these task differences has been that ambiguous words generate a noisy semantic code that facilitates LDT (e.g., Hino and Lupker, 1996). In contrast, in semantic tasks the noisy code must be resolved to some degree in order to respond, and this takes time, often delaying responses to ambiguous words (e.g., Hoffman and Woollams, 2015).

While these dissociations between ambiguity effects (for SemD and other ambiguity metrics) have been observed across tasks for nouns in previous research, here we observed the dissociation for verbs within a single task. Our assumption is that in the past-tense generation task, responses to irregular verbs require more extensive semantic processing than do responses to regular verbs (Butler et al., 2012). The semantic processing involved in generating the past tense of irregular verbs is slowed by high SemD. Thus, while high SemD tends to be an advantage for generating the past-test forms of regular verbs, it is a disadvantage for irregular verbs.

In a recent megastudy, Cohen-Shikora et al. (2013) examined the effect of imageability on past tense generation latencies for a larger set of verb stimuli (2200 items), only some of which overlap with the present, smaller set of items.^3^ They found that imageability was a significant predictor of past tense generation latencies, after onsets, frequency, and orthographic characteristics had been controlled. In their analysis, Cohen-Shikora et al. (2013) did not examine other semantic variables and, importantly, did not examine interactions with regularity. In contrast, Butler et al. (2012) did test the interaction of imageability and regularity in past tense generation and found that imageability effects were significantly larger for irregular verbs than for regular verbs. Further, Butler et al. (2012) found that semantic priming influenced past tense generation latencies for irregular verbs but not for regular verbs. The results of both of these previous studies are consistent with our inference that semantic richness effects can be observed for verb stimuli. Further, it has been argued that the particular pattern of semantic effects observed in past tense generation tasks will depend on factors such as list context (Cohen-Shikora and Balota, 2013), and other aspects of task demands (Woollams et al., 2009). Our findings, and the differences between our findings and those of previous studies, can be taken as support for this inference.

In Experiment 2, in addition to regularity, we explored another distinction that is only relevant to verb meaning; we manipulated verb tense and examined effects on ratings of embodiment. We found that embodiment ratings were modulated by an interaction of tense and regularity: ratings tended to be lower for irregular verbs presented in past tense than in present tense, suggesting that tense is relevant to verb meanings, even those derived in simple ratings studies. When making embodiment ratings, participants are asked to judge how easily the human body is involved in the action, state, or relation referenced by the verb. Thus, they consider the extent to which the human body is involved in the verb’s meaning. Our results suggest that they consider the body to be more easily involved in *sit* than in *sat*. If this is true, one prediction for future research would be that if verbs were presented in the past tense in LDT, lexical decision latencies would not be affected by embodiment to the same extent as they were in the present Experiment 1, where verbs were presented in present tense.

An important caveat is that differences in frequency seem to contribute to the effects of tense on embodiment. These patterns should be explored more fully in future research. To the extent that verbs do differ in embodiment in their present and past tense forms, this would be consistent with studies on temporal construal level, which have found that the farther away an event is temporally, the more likely it is to be represented in terms of its abstract features (Trope and Liberman, 2003). These abstract, high-level construals include general features capturing the essence of the event, but omit concrete and incidental details. Thus, when imagining the extent to which the body is involved in an event taking place in the past, sensorimotor features may be less pronounced, leading to lower ratings. Indeed Trope and Liberman (2010) theorized that as the distance to event increases, that event’s representation will include less multimodal simulation, and more amodal symbolic representation.

An interesting potential to emerge from Experiment 2 was that the difference between embodiment ratings for verbs in the present and past tense tended to be observed for irregular as compared to regular verbs. One explanation might be that the transparent morphology of the regular past tense verbs made the verb stem (the present tense form) more salient, attenuating the effect of temporal distance on embodiment ratings. Another explanation could be based in the frequency difference for regular and irregular verbs: irregular verbs tended to be more common (as measured in their present tense form, though this also holds true when in their past tense form), and this familiarity may have prompted more vivid simulations for irregular verbs. These more vivid simulations could produce the higher relative embodiment ratings we observed for irregular verbs in general, and could also have been more strongly affected by the effects of temporal distance. Clearly this is a question that requires further research to fully explore.

In the present study, there were several differences observed between regular and irregular verbs. Our understanding of some of these can be informed by previous research. For instance, research has demonstrated that the more frequently an irregular verb is used, the more resistant it is to regularization (i.e., adopting a regular *-ed* ending; Lieberman et al., 2007). This helps clarify why irregular verbs had higher mean frequency than regular verbs in our sample – these are the irregular verbs that have survived regularization by virtue of their frequent use. Other differences are more difficult to explain, such as the higher valence and embodiment of irregular verbs. It might be informative to examine differences in the historical origins of regular and irregular verbs. Many irregular verbs can be traced back to a system of conjugation in Old English in which “strong” verbs were conjugated by way of a stem vowel change (e.g., *grinde* to *grand*, the ancestor of *grind/ground*; Lieberman et al., 2007). This was contrasted with “weak” verbs, which took on variants of the suffixes *–t* or *-d*, the origin of Modern English’s regular past tense inflection (Hare and Elman, 1995). Interestingly there were some differences in which kinds of verbs belonged to either system. Verbs created out of nouns or adjectives tended to take on the “weak” verb inflection, as did verbs adopted from other languages; conversely many “strong” verbs can be traced back to Proto-Germanic. Of course in the intervening millennia many verbs have shifted from one type of inflection to another, and it is certainly true that not all irregular verbs followed the “strong” pattern of inflection. Nevertheless future research might examine if these historical factors contributed to some of the differences observed in this study.

Certainly, there are limitations to the present study. Our analyses were focused on dimensions that seemed to be likely candidates for verb meaning and for which we had values for a reasonable number of verbs, in order to facilitate item-wise regressions. We also limited our investigation to semantic dimensions that could theoretically apply to all verbs. We did examine effects of verb regularity and tense, but there are other distinctions one can draw between types of verbs. As such, there are other dimensions and distinctions that should be examined in future studies (see, e.g., Gennari and Poeppel, 2003, for discussion of other distinctions and dimensions that may be important for verbs).

We treated regularity as a binary category in the present study, as a convenient way to explore potential differences in semantic richness effects. This is certainly not the only possible approach to coding the quasi-regularities involved in English past tense inflection (e.g., Woollams et al., 2009), but the results provide insight about differences in semantic richness effects for regular and irregular verbs. As mentioned, there is considerable debate about past tense inflection of regular and irregular verbs (e.g., Pinker and Ullman, 2002; Joanisse and Seidenberg, 2005), and our results do speak to that theoretical issue. That is, while some models of past tense inflection (e.g., Pinker and Ullman, 2002) suggest that lexical knowledge is the main driver of past tense generation, our results suggest, instead, that semantic knowledge is important when generating the past tense for irregular verbs. Thus, our findings are more consistent with models that suggest semantic knowledge is the more influential factor in explaining irregular past tense inflection (e.g., Joanisse and Seidenberg, 1999; Woollams et al., 2009).

## Conclusion

The present results provide evidence that the semantic richness approach can be extended beyond nouns, and that much can be learned about verb meaning by examining the effects of semantic richness variables, even when verbs are presented in isolation. While certain factors particular to verbs such as tense and regularity may make this somewhat more complicated, they may also provide fruitful avenues for future research. Our findings suggest that verb meaning is multidimensional, and that the effects of different aspects of verb meaning will depend on task demands.

## Author Contributions

DS designed and ran all experiments and collaborated on manuscript writing. AH analyzed some of the data and collaborated on study design and manuscript writing. PP collaborated on study design, data analysis, and manuscript writing.

## Conflict of Interest Statement

The authors declare that the research was conducted in the absence of any commercial or financial relationships that could be construed as a potential conflict of interest.

## Footnotes

1 We ran the same regression analysis, but included dominance instead of valence; the relationship between dominance and LDT latencies was significant, *sr* = -0.10, *p* < 0.01. We also ran the same regression analysis, but included imageability instead of embodiment; the relationship between imageability and LDT latencies was not significant, *sr* = -0.04, *p* = 0.35.

2 www.surveymonkey.com

3 Of the 422 verbs used in the present study, 238 were also included in Cohen-Shikora et al.’s (2013) Past Tense Inflection Project (PTIP). For these items, the correlation between past tense generation latencies across the two data sets was strong, *r*(238) = 0.80, *p* < 0.001.
