# Supplementary material for: Is More Always Better for Verbs? Semantic Richness Effects and Verb Meaning
Source: Front Psychol. 2016 May 31;7:798. doi: 10.3389/fpsyg.2016.00798 (PMC4885847; doi:10.3389/fpsyg.2016.00798)
Supplement: Supplementary file 1 [file Data_Sheet_1.DOCX]

**Appendix**

Instructions for Embodiment Ratings

Verbs are words that typically express an action, state, or relation between two things. The meanings of many verbs refer to actions, states, or relations that easily involve the human body, whereas the meanings of other verbs refer to actions, states, or relations that do not easily involve the human body. For example, the meanings of verbs such as “to leap” and “to sleep” easily involve the human body, whereas the meanings of verbs such as “to appreciate” and “to dissolve” do not easily involve the human body. Any verb (e.g., “to leap”) that in your estimation refers to an action, state, or relation that easily involves the human body should be given a high rating (at the upper end of the numerical scale). Any verb (e.g., “to appreciate”) that in your estimation refers to an action, state, or relation that does not easily involve the human body should be given a low rating (at the lower end of the numerical scale). It is important that you base these ratings on how easily an action, state, or relation involves a human body and not on how easily it can be experienced by human senses (e.g., vision, taste, etc). Also, because words tend to make you think of other words as associates, it is important that your ratings not be based on this and that you judge only how easily an action, state, or relation involves a human body.

The purpose of this experiment is to rate verbs regarding how easily an action, state, or relation involves a human body. In other words, how important is having a body to understanding the meaning of each verb?

Your ratings will be made on a 1 to 7 scale. A value of 1 will indicate actions, states, or relations that do not easily involve the human body, and a value of 7 will indicate actions, states, or relations that do easily involve the human body. Values of 2 to 6 will indicate intermediate ratings. Please feel free to use the whole range of values to make your ratings. When making your ratings, try to be as accurate as possible, but do not spend too much time on any one word.
